# Ethical challenges and action alternatives: Case reflections in ambulance care

**DOI:** 10.1177/09697330251403138

**Published:** 2025-12-03

**Authors:** Anna Bennesved, Anders Bremer, Anders Svensson, Andreas Rantala, Mats Holmberg, Joar Björk

**Affiliations:** 1 Faculty of Health and Life Sciences, 4180 Linnaeus University, Växjö, Sweden; 2 Centre of Interprofessional Collaboration Within Emergency Care (CICE), 4180 Linnaeus University, Växjö, Sweden; 3 Department of Ambulance Service, Region Kronoberg, Växjö, Sweden; 4 Department of Health Sciences, Faculty of Medicine, 5193 Lund University, Lund, Sweden; 5 Department of Ambulance Service, Region Skåne, Helsingborg, Sweden; 6 Centre for Clinical Research Sörmland, 8097 Uppsala University, Eskilstuna, Sweden; 7 Department of Ambulance Service, Region Sörmland, Katrineholm, Sweden; 8 Centre for Research Ethics & Bioethics, Department of Public Health and Caring Sciences, 8097 Uppsala University, Uppsala, Sweden; 9 Department of Research and Development, Region Kronoberg, Växjö, Sweden

**Keywords:** ambulance clinicians, caring, decision-making, ethics case reflections, patient autonomy

## Abstract

**Background:**

Ambulance clinicians regularly encounter medical, caring, existential and ethical challenges. Meeting patients with complex medical presentations underscore the need for holistic decision-making and actions as ambulance clinicians struggle to strike a balance between addressing medical and caring needs.

**Aim:**

This study aimed to explore action alternatives considered and discussed during ethics case reflections in response to care-related challenges in ambulance services.

**Research design:**

A qualitative descriptive study design was applied. Data were analyzed using conventional and summative content analysis.

**Participants and research context:**

Ethics case reflections were performed with 14 groups comprising a total of 78 ambulance clinicians. Prior to the reflections, a video depicting the encounter between two ambulance clinicians and an older patient and his spouse was viewed.

**Ethical considerations:**

The principles of the Declaration of Helsinki were applied throughout the research process, and an advisory statement was obtained from the Swedish Ethical Review Authority (No. 2019-02127 and 2021-03490).

**Findings:**

The ethics case reflections generated a variety and breadth of action alternatives to manage challenges in caring, suggesting that this format is suitable for discussing ethical issues in clinical cases that depart from standard medical emergencies. Furthermore, the breadth of the results reveals the wide professional discretion afforded to ambulance clinicians and suggest the presence of tacit competences embedded in professional practice.

**Conclusions:**

Ethics case reflection has a potential to enhance ambulance clinicians’ ethical decision-making by deepening reflections about patient autonomy as well as highlighting the potential for a caring approach and promoting holistic care. By generating a breadth of specific action alternatives, many possible ways forward even in situations with complex care-related challenges are illustrated. Further investigation regarding the role of ethics case reflections to articulate implicit attitudes and tacit competencies is warranted.

## Introduction

Ambulance clinicians (ACs) regularly encounter medical, caring, existential, and ethical challenges. Assessing patients with complex medical presentations highlights the need for holistic decision-making, as ACs strive to balance immediate medical needs and interventions with caring needs and nursing actions. This study therefore focuses on exploring the action alternatives considered in ambulance services (ASs) when navigating such challenges.

## Background

Ambulance services provide prehospital care in a constantly evolving landscape shaped by societal, medical, ideological, and organizational changes. ACs are highly trained for assessing whether a patient’s condition is medically urgent, time-critical, or non-urgent—considering factors such as self-care capacity. Their work involves navigating medical, caring, existential, and ethical dimensions, as patients’ suffering may be physical, psychological, existential, or a combination of these.^1^

Patient assessment raises ethical concerns, as ACs must interpret clinical signs while also seeking to understand the patient’s lifeworld.^2^ To provide holistic care, ACs must adopt a caring approach that gives patients a voice by incorporating their narratives into the care process.^3^ This approach requires deep human understanding and the ability to navigate the interplay between objectivity and subjectivity. Rather than viewing professional detachment and attention to patients’ lived experience as opposing forces, ACs should integrate both perspectives in a trust-based relationship. Such an approach involves a continuous movement between them, ensuring that medical treatment is integrated with the patient’s sense of self and well-being. Overcoming these challenges demands not only interdisciplinary collaboration but also a shared language between the patient and the healthcare professional that bridges medical and experiential knowledge.^4^

In addition to other competences, ACs must also have ethical competence.^5,6^ One particular skill is responding to the healthcare needs of older patients,^7^ who often present with non-urgent conditions.^8,9^ These situations require ACs to assess decision-making capacity while respecting patient preferences, which is ethically challenging.^2^ Ethical guidelines emphasize involving older patients in decision-making whenever possible, and empirical evidence suggests that they value this involvment.^10^

Although the emergency department is often the primary destination, both ACs and patients may sometimes prefer non-conveyance or referral to primary care.^11,12^ However, non-conveyance decisions can be fraught with different expectations and conflicting perspectives.^13,14^ While guidelines exist to support ACs in making these decisions, they are often perceived as insufficient due to their limited specificity and applicability. In many cases the guidelines fail to sufficiently reflect the nuanced clinical judgment required in prehospital settings. The guidelines offer limited support in navigating the delicate balance between respecting patient autonomy and managing clinical risk.^15^ Situations involving non-urgent medical needs—especially when combined with social or ethical complexity—can be just as challenging as medically urgent cases.^16,17^

Previous studies highlight the need for structured ethical reflection to support ACs in navigating ethically complex situations, particularly when caring for patients with impaired decision-making capacity. Ethics Case Reflection (ECR) is one such method used in healthcare to facilitate dialogue and critical thinking around ethically difficult situations.^18–20^ Although ECR has not yet been extensively studied within AS, it has been applied in other healthcare contexts, including pediatric oncology^21^ and hospital-based care.^20^ In these settings, ECR has proven to be a valuable tool for enhancing team cohesion, clarifying ethical concerns and supporting clinicians in ethically challenging situations. For instance, the ECR format allows for detailed discussions of proxy decision-making, balancing respect for autonomy with consideration of best interests, and fostering consensus around ethically sound care plans when interacting with patients with limited capacity for self-determination.^21,22^

Building on this, the present study aimed to explore action alternatives considered and discussed during ethics case reflections in response to care-related challenges in ambulance services.

## Methods

### Design

A qualitative descriptive study design was applied. Data were analyzed using a combination of summative^23,24^ and conventional content analysis^25^ to explore data from ECRs.^19^

### Recruitment and participants

Through purposeful sampling in three Swedish regions with a combined population of approximately 750,000 inhabitants, potential participants (*n* = 346) matching the inclusion criterion were identified. Being a clinically active AC at the time of data collection constituted the inclusion criterion. Eligible participants received both written and verbal information about the study. They were informed that participation was voluntary and that consent could be withdrawn at any time prior to the ECR session. Participants self-registered for a session of their choice and provided informed consent at the time of registration. To ensure that all potential participants received information about the study, unit managers reminded eligible staff verbally during staff meetings.

In total, 78 individuals (23%) agreed to participate (Table 1). Fourteen ECR groups were formed—eight conducted face-to-face at participants’ workplaces and six held digitally via Zoom—with three to seven participants per group (median = 6).

**Table 1.** Demographics of the participants (*n* = 78).

Sex	
Male/female	39/39
Age (years)	
Median	42
Min–Max (range)	24–65
Education	
RN/EMT^a^	74/4
RN with specialist education^b^	60
Work experience in AS (years)	
Median	10
Min–Max (range)	0.5–34

^a^RN = Registered Nurse (3 years of education, master’s level), EMT = Emergency Medical Technician (2 years education, upper secondary level).

^b^Prehospital Emergency Care, Public Health Care, Emergency Care, Intensive Care, Anesthesia Care (all are 1 year of specialist training, master’s level).

## Data collection

### The video case used in the ECRs

A 25-min video (Table 2) initiated the ECR discussion. The video was studio-recorded for this project. Professional actors played the roles as the patient and his wife, and experienced ACs played professional roles. The script was written to illustrate an ethically salient situation in the prehospital setting based on previous studies, namely, how to address patient autonomy involving patients with potentially impaired decision-making capacity in non-urgent medical situations.^26,27^ The video case did not specify the presence of particular ethical challenges. Instead, it was designed to allow participants to interpret what they considered ethically relevant. The scenario naturally raised questions about autonomy and the relationships between the patient, his wife, and the ACs. Some ethical challenges were more readily addressed through this case, while others—such as side effects, hierarchies, and threats—were less prominent or relevant in this context.

**Table 2.** The roles and plot in the recorded video case.

The patient	• An elderly man
	• He possibly has impaired decision-making capacity related to an unknown cause
	• He refuses to be conveyed to the hospital
The wife	• Slightly younger than the patient
	• She appears stressed
	• Her cognitive function is unclear
	• She wants the patient to be conveyed to the hospital
The ambulance clinicians	• Two trained/licensed nurses
	• The ACs in the video demonstrate different interaction styles: One is calm and empathetic, while the other appears more stressed and matter-of-fact
	• The ambulance clinician is unsure whether the patient should be conveyed to the hospital
The plot	The patient experienced abdominal pain during the night, prompting concern from his wife about his health. She calls for an ambulance. While arriving at the scene the ambulance clinicians (ACs) interact with both the patient and his wife during the physical examination. Comprehensive interviews reveal that the patient suffers from anxiety, loneliness, and impaired memory. The physical examination showed no alarming medical signs, indicating that the situation was not urgent. The patient was offered a hospital visit for further evaluation but strongly declined. However, the interview did not clarify what type of care the patient preferred or why he refused hospital treatment. The video ends before the visit is concluded, at a point where the ACs have yet to decide how to proceed with the situation

### The ECRs

All ECRs were conducted in accordance with the 6-step model (6SM),^19^ and facilitated interactively, lasting between 74 and 99 min (mean duration: 85 min). The sessions were led by the last author, an experienced clinical ethicist, who guided the group through the six steps using a collaborative, group-work approach (Table 3). Although the steps were generally followed in sequence, participants were allowed to move back and forth between steps as needed. The facilitator summarized the group’s input in real time using a worksheet visible to all participants. All ECRs were audio-recorded using digital equipment and transcribed verbatim by a professional transcriber.

**Table 3.** The 6-step model for ethics case reflections.

Stage I	What is the ethical challenge?
Stage II	Which are the relevant medical and social facts of the case?
Stage III	Who are the parties involved, and what are their views and interests?
Stage IV	Which values, principles and legislation may be of relevance in the situation?
Stage V	What are the possible action alternatives?
Stage VI	Holistic discussion/evaluation of courses of action

## Data analysis

The data consisted of “possible action alternatives” generated during Stage V of the 6SM (Table 2). In this stage, participants were asked to suggest potential action alternatives that could be employed by some person present in the video. For the sake of discussion even controversial or unusual alternatives were invited. A conventional content analysis was applied to the transcripts to gain a deeper understanding of participants’ discussions.^25^ Initially, 211 action alternatives were identified and coded based on content. Codes were then compared and consolidated by identifying overlaps and redundancies,^28^ resulting in 134 unique action alternatives. These were grouped into subcategories and categories. While coding primarily followed a manifest content approach, contextual interpretation was occasionally applied to ensure accurate categorization. In parallel with the conventional content analysis, a summative content analysis^23,24^ was adopted to determine the frequency and occurrence of action alternatives both within individual ECRs and across the dataset.^23^

In presenting findings, “participants” refers to study contributors, while “ACs” denotes ambulance clinicians in general or those depicted in the video. This study was documented and reported in accordance with Standards for Reporting Qualitative Research (SRQR).^29^

## Ethical considerations

The study was conducted according to the principles of the Declaration of Helsinki,^30^ and an advisory opinion was obtained from the Swedish Ethical Review Authority (No. 2019-02127 and 2021-03490).

## Findings

The following section presents the combined results from the 14 ECRs. Findings are organized into categories and subcategories, listed in descending order of frequency. To enhance clarity and contextual understanding, representative action alternatives are included throughout. This is followed by the results of the summative content analysis, which shows the frequency and distribution of the 134 identified action alternatives. In the sections below, the term “action alternatives” is occasionally abbreviated to “alternatives.” The alternatives were grouped into four overarching categories (Table 4).

**Table 4.** Categories and subcategories.

Category	Subcategory
Decision on conveyance	1. Conveyance with coercion
	2. Non-conveyance with plan
	3. Conveyance without overt coercion
	4. Rapid non-conveyance
	5. Non-conveyance with advice
Before conveyance	6. Assessment of preferences
	7. Explaining the situation and options available
	8. Obtaining medical and social information
	9. Interacting with patient and family members separately
	10. Seek decision support
Quality of AC team interaction	11. Need for improved interaction in patient encounters
	12. Need for improved interaction before and after the patient encounter
Ideas and ideals	13. Inspiration from caring ideals
	14. Improved help from other healthcare professionals
	15. Improvements to the ambulance organization

## Decision on conveyance

The alternatives in this category implied that a decision on conveyance had already been made. Subcategories were based on the premise that the patient should *not* be conveyed by ambulance to hospital or PHC whereas two subcategories implied that the best course of action was to transport the patient to either ED or PHC. As the patient in the video refused the suggestion to be conveyed, these alternatives presented strategies for changing the patient’s mind or conveying regardless of changed opinions.

### Conveyance with coercion

Participants also described unambiguous instances of coercion. For instance, there were clear cases of physical coercion (e.g., “carry the patient out against his will”), implying that the patient had no other option than to accept conveyance (“now you’ll just have to get on the stretcher, there is no choice”) or aggressive language (“get in the ambulance or get lost”).

### Non-conveyance with plan

Rather than just giving recommendations, the ACs could create a plan for patients. Many alternatives revolved around the PHC: “ask the GP to make a home visit” and “book an appointment at the PHC.” There were also more indirect plans: “contact the PHC and tell them that it is unclear whether he takes his medications” and “make it clear in the patient journal that there are social problems here.” These alternatives likely reflect the ACs’ unique insights gained from observing the patient in their home environment—insights that others may lack but could benefit from.

Although most plans involved a medical check-up, some plans involved the patient’s family members. This means that family members could play a beneficial role if they receive relevant information and encouragement.

### Conveyance without overt coercion

ACs could help change patients’ minds so that they can be conveyed by an ambulance by engaging in a longer conversation and using deception or distraction. For instance, it was suggested that ACs choose their words to suggest that things were already settled (“so you bring your phone and I’ll take the charger”) or frame the patient’s options as a dichotomy (“it’s all or nothing”). Another suggestion was to scare the patient compliant (“frighten by saying: you don’t want to end up like *them*, do you?”, alluding to the death of two loved ones mentioned in the video) and appealing to the patient’s sense of guilt and responsibility (“surely you don’t want to cause trouble for your wife…?”).

It was also suggested that the wife or the patient’s children be encouraged to influence the patient to come along. Loved ones may have greater leverage over the patient than ACs and that ethical and legal rules that prohibit ACs from pressing patients do not apply to family members. Collectively, all alternatives aimed at helping patients change their minds and accept conveyance.

### Rapid non-conveyance

The alternatives in this subcategory all implied that it was obvious from the video that no or little intervention was needed as implied by the alternative “give pain relief and go,” and that it would be unnecessary to spend more time with the patient. These alternatives thus communicated a sense of urgency—staying with the patient means, in a sense, time lost. There were two reasons for rapid non-conveyance: First, there appeared to be no medical urgency to the case, or second, the patient stated that he did not want to accompany the ACs to the hospital. This was clearly shown in the alternative, “as soon as the patient says no—go.”

### Non-conveyance with advice

The alternatives here implied that the ACs could improve the situation by providing advice to the patient and/or his wife. Many of these recommendations explicitly or implicitly placed responsibility on the patient and/or his wife to seek care elsewhere or later (e.g., “encourage the wife to call the PHC” and “tell them to call again if it gets worse”). Some alternatives focused on the patient’s social and existential situation (e.g., “get involved in social activities”).

## Before conveyance

The common feature in this category was the suggestion that more actions could be carried out before deciding to convey or not convey the patient.

### Assessment of preferences

Several alternatives highlighted the importance of exploring patient preferences. For example, the alternative “ask what the patient truly wants; going beyond first impressions” encourages a deeper understanding of the patient’s thoughts, fears, and reasons for declining conveyance. These alternatives suggested an exploratory approach to gathering information, including probing and in-depth questions, to reach the core of the patient’s preferences. Several alternatives also suggested assessing the wife’s preferences in a similar fashion.

### Explaining the situation and options available

The alternatives in this subcategory implied that the patient and his wife may not comprehend the situation and possible options and that the AC could aid by providing specific information. In this way, the patient may be helped to make an informed decision. For instance, the AC could explain the different options for medical assistance. It was suggested that the patient could be informed of the possibility of visiting the hospital by taxi. It was further suggested that creating a decision tree could enhance the patient’s understanding of the situation and help prevent misunderstandings (“explain that going to the hospital would not mean staying there forever”). ACs could also express their own assessment of the situation (“tell them what I see, including what seems not to be working”).

### Obtaining medical and social information

Suggested alternatives related to obtaining more information than what was already presented in the video. Alternatives described practical means of gathering information, whereas others described questions about the patient’s previous or present medical concerns, which could be presented to the patient, the wife, other members of the family or other caregivers. It was suggested that the medical records be read (Swedish ACs generally do not have immediate access to this information), either using a computer in the ambulance or calling the hospital. More information could be collected regarding the patient’s stomach pain, his sleeping habits and his cognitive status. To create a broader perspective of the patient, including his social situation, it was suggested that information could also be collected from family members and by observing the home environment (e.g., in the alternative “look for a folder from the home health care service”).

### Interacting with patient and family members separately

It was suggested that the ACs speak to the patient and his wife separately to increase the chances of receiving valuable information and/or improving the interaction (“make sure to split them up to assess their separate points of view”).

### Seek decision support

In this subcategory alternatives have focused on AC requesting help in decision-making. Often this idea took the form of asking for help from a physician, although alternatives suggested that the wife could be a source of decision support. The help sought was advice on decision-making (“call a Primary Healthcare Centre [PHC] or ED physician to get advice about conveying or leaving at home”) or legal/moral support.

## Quality of AC team interaction

This category focus on the interaction within the AC team, providing suggestions for what the ACs shown in the video could do to improve their communication and interaction with the patient and his wife. Although this may of course be in the patient’s interest, alternatives were also mentioned as being in the ACs’ own interest.

### Need for improved interaction before and after the patient encounter

Alternatives provided suggestions for suitable conversations within the AC team before the patient’s home was admitted. Phrases like “let’s try to take time to listen to what they want” and strategies like “talk things through in advance, even in this kind of situation” (alluding to the standard mental preparation for medical emergencies) helped create a shared understanding. Also providing feedback after the participants left the home, ranging from explicit feedback (“I think your attitude just wasn’t okay”) to understated feedback (“perhaps we could have done that better—perhaps we should have warned the patient when we checked his vitals because he seemed to dislike that”).

### Need for improved interaction in patient encounters

Participants suggested ways that one AC could influence his/her colleague to act differently. The examples included both direct communication (“now stop it” and “let’s start this over”) and indirect communication such as exaggerating one’s own good behavior to influence the colleague or saying “oops, that hurt” during the examination to signal empathy to the patient as well as warning the colleague that he/she was too overbearing.

## Ideas and ideals

This category addressed improvements in the quality of care.

### Inspiration from caring ideals

The ECRs included discussions about the attitudes expressed by the ACs in the video, along with alternatives that illustrated more caring communication with the patient. This would express respect for the patient and/or his wife and provide space for him (and her) to be seen and heard. Examples of alternatives included “put words to the wife’s situation: you carry a heavy load—this will strengthen her” and “make them feel heard somehow, don’t just say no.” Moreover, some suggestions were of a general nature, such as “go beyond the medical to the deeper aspects” and “focus on the medical first and the social afterwards.”

### Improved help from other healthcare professionals

Few potentially desirable alternatives that were not available in the video should be made available in the future. For example, it was suggested that ACs should have easy access to a geriatric team or a home care physician who could be called to the patient’s home while the ACs were still there.

### Improvements to the ambulance organization

How ACs are trained, the way in which ACs approach and discuss cases could be improved. For instance, the statements “management should assert that these patients, too, are our responsibility” (i.e., in addition to medically urgent cases) and “raise the status of this kind of patient” suggested that there may be negative or dismissive attitudes about some of the ACs’ tasks. It was suggested that ACs could benefit from reflection groups or specific training to handle non-urgent medical situations. Several alternatives also highlighted that it may be relevant to consider how AC teams are composed, referring to teams where both ACs have an uncaring attitude.

## Summative analysis of the action alternatives

**Figure 1.**
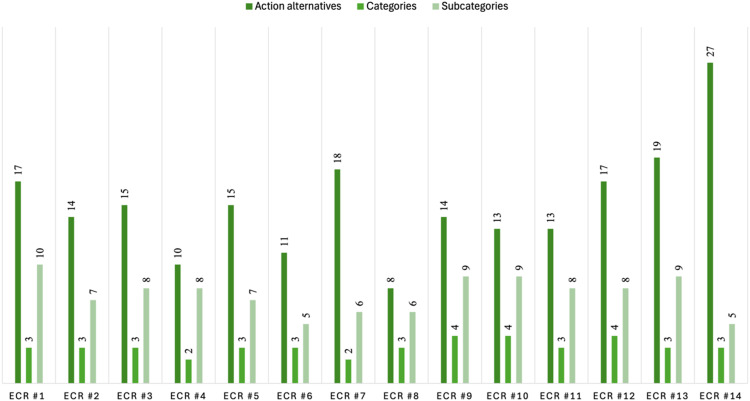
Number of action alternatives and number of categories/subcategories represented per ethics case reflection.

The ECR groups showed considerable variation in the number of alternatives generated. With respect to the ECR with the fewest alternatives (ECR # 8), eight alternatives were generated, whereas the most prolific group (ECR # 14) generated 27 alternatives (M = 15/ECR) (Figure 1).

**Figure 2.**
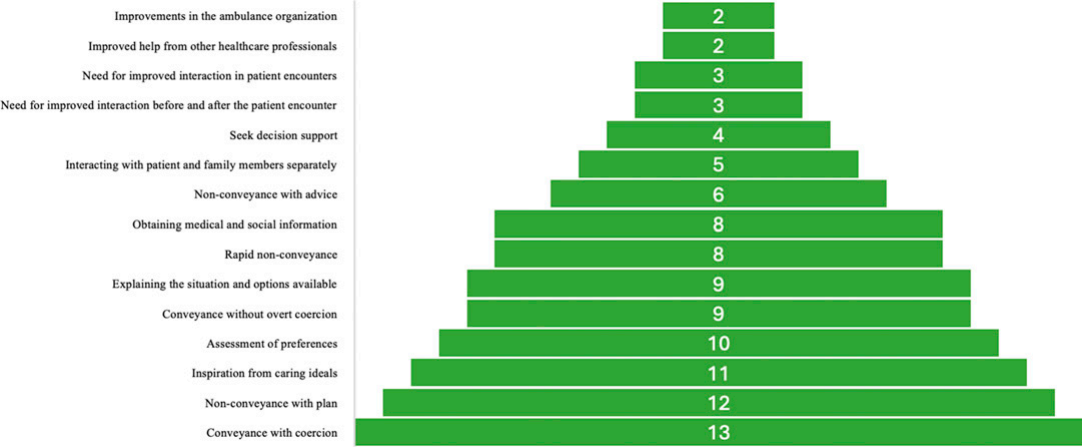
Number of ethics case reflections in the subcategories.

The most frequent alternative *(Conveyance with coercion)* was present in 13 out of 14 ECRs. The second most common alternative *(Non-conveyance with plan)* was present in 12 of the 14 ECRs, and the least common alternatives *(Improvements to the ambulance organization* and *Improved help from other healthcare professionals)* were present in two ECRs (Figure 2).

No ECR featured alternatives from all 15 subcategories. The greatest number of subcategories was 10 in ECR1, followed by nine in ECR9, ECR10, and ECR13 (Figure 1). For the four main categories, three of the 14 ECRs contained alternatives from all four categories, nine of the 14 ECRs contained alternatives from three of the four categories, and two of the 14 contained alternatives from two of the four categories. No ECR contained alternatives from only one category.

## Discussion

The action alternatives presented by experienced ACs provide novel and important insights into the ethical issues when facing care-related challenges, as well as into ECRs as a mode of working with these issues and challenges. As seen in the results section, participants perceive the main ethical dilemma in the video to revolve around the patient’s autonomy and how his wishes align with his best interests.

Autonomy is widely identified as a fundamental ethical principle in healthcare,^31^ and protected as a human right by international declarations and conventions.^32,33^ While autonomy is inherent to all individuals, the implications of autonomy differs between situations. Within AS, autonomy should be conceptualized as a relational and context-dependent ethical principle.^34^ The demands of autonomy must also be balanced against the demand to act in accordance with beneficence. In situations requiring immediate intervention, the urgency to prevent harm may limit the possibility for informed consent or patient reflection. However, in care situations where there is more time for dialogue and deliberation, there is an opportunity to work with the patient and his autonomy.^35^ Previous studies from the AS setting highlight how patients autonomy can be impacted by entering residential care,^36^ becoming dependent on others,^37^ approaching the end of life, and experiencing conflicts between patients and significant others.^16^ Our results add to this knowledge in several ways. First, it supports previous findings that ACs find it particularly difficult to respond to autonomy challenges vis-à-vis older patients with impaired decision-making capacity when clear medical incentives are lacking.^2^ One implication is that this area demands more attention in AC education. Second, previous literature has suggested that context-sensitive decision-making in the AS can be supported by using practice-based models for reflection,^16^ and/or that ethical competence can be strengthened through education, particularly in terms of autonomy and trust.^26^ Our results suggest that conducting ECRs can be a valuable complement to ethics education. Using a case such as the one in this study may support what has been described in previous research^38^ as an opportunity for reflection and dialogue grounded in real-life situations. The ECR method provides a structured yet flexible format that encourages learners to explore ethical dimensions without predetermined answers. This approach fosters reflective practice and enhances ethical awareness, making it a practical and meaningful tool for ethics education. Third, texts on relational aspects of autonomy emphasize that the capacity for autonomous decision-making is deeply shaped by interpersonal relationships, trust, and communicative practices.^39^ Autonomy, from this perspective, is co-constructed through social interaction and shaped by the relational contexts in which individuals find themselves. By acknowledging the perspectives of those close to the patient, ACs can gain a nuanced understanding of the patient’s situation, values, and preferences.^34^ Our findings indicate that ACs are attracted to a relational approach, as several alternatives involved the patient’s wife in this ethically complex situation.

Another important aspect of our findings is the broad and dynamic caring competencies found within AC work. Several ECRs exposed a range of well-integrated medical, social, and relational approaches to managing the situation, reflecting a nuanced understanding of the patient as a person with complex needs and their own lifeworld. In line with Merleau-Ponty’s philosophy, which views the human being as an inseparable unity of body, soul, and world,^40^ the participants’ responses demonstrate that care is not merely a technical act but a relational and existential practice. This suggests that at least in the ECR, ACs can articulate highly complex ideals of professional nursing practice.

By synthesizing the results of all 14 ECRs, the study provides insight into the many strategies that may be employed in complex, non-urgent situations. While some alternatives focus narrowly on biomedical concerns, others broaden the scope to include social and relational dimensions of care. The involvement of the patient’s wife, consideration of the patient’s previously expressed wishes, attention to personal resources within the patient’s lifeworld and the simultaneous interpretation of medical and existential dimensions exemplify profound caring competences as described by the EXPAND-model.^3^

The suggested alternatives are remarkably heterogeneous, highlighting the potential for various approaches in non-urgent cases. Also, the results illuminate embedded caring competences inspired by caring ideals, such as providing empathic understanding and confirmation in complex nursing situations.^41^ By partaking of the alternatives, we also understand the skills and know-how necessary to even come up with such alternatives. To suggest actions such as arranging for a geriatric team to visit a patient at home or contacting a physician who can speak with the patient by phone, participants need to have contextual knowledge of the healthcare resources available. They also need to understand how communication with various stakeholders may influence the patient’s experience and outcomes. On the other hand, implementing approaches like using an informative rather than persuasive communication style, or fostering trust, requires strong interpersonal skills, particularly in communication and relationship-building.

In the following section, the results are presented as a reflection on the professional competence of ACs, guided by Aristotle’s concept of *phronesis*—practical wisdom. Applied to healthcare professionalism, phronesis involves discerning the right course of action for a particular individual in a specific situation.^42^ It is a form of knowledge cultivated through experience, reflection, and ethical sensitivity, rather than something innate or automatic.^43^ Furthermore, phronesis should not be seen solely as an individual trait but as a collective and contextual capacity that is realized in interaction with others. It is through dialogue with colleagues, reflection on past experiences, and encounters with patients’ unique situations that practical wisdom is developed.^42^ The results in this study shed light on embedded caring competences inspired by caring ideals, such as providing empathic understanding and affirmation in complex situations,^41^ previously described as tacit competences,^44^ which are generally hard to elucidate. The scenario, portrayed in the video, illustrates a range of care-related challenges that require a high degree of sensitivity to contextual details and the ability to perceive the uniqueness of the situations. These are qualities that align with a phronesis-based understanding of competence. It may be tempting to interpret the action alternatives as a matter of “common sense,” as if the right course of action were obvious or intuitive. However, phronesis is in fact a highly specialized form of practical wisdom which becomes especially evident in a situation where the rules give no clear guidance and ACs must act in a novel way according to the unique situation.^45^ The breadth of perspectives evident in this study can be understood as an expression of phronesis as it reflects an awareness that multiple responses may be appropriate in a given situation, and that combining approaches is often more effective than relying on binary, either/or thinking. Striving to combine different approaches may be extra important in the “grey zones” of healthcare, where needs, perspectives, and values often coexist in tension.^46^ Participants in our study advocated a “building blocks” approach to alternatives. This refers to a type of reasoning where participants propose a general alternative with minimal detail and then elaborate on it by suggesting various ways it could be implemented. For example, the idea of leaving the patient at home might be expanded with additional actions such as scheduling a follow-up visit or offering emotional support to a family member. This “building blocks” approach can be seen as a highly contextualized method for resolving points of conflict that arise in complex care situations.

## Methodological considerations

### Strengths

A key strength of this study is its use of a video case, which distinguishes it from previous ECR studies. While some ECR approaches rely on participant-generated cases, the use of a pre-recorded video is fully compatible with the 6-Step Model (6SM).^19^ Viewing a video may have fostered greater participant engagement than a verbally presented case, although it remains unclear whether this influenced the nature or number of action alternatives proposed. Importantly, unlike cases designed to highlight specific ethical dilemmas, the video used in this study did not explicitly present a defined ethical challenge. Instead, it invited participants to interpret and identify ethically relevant aspects themselves. For instance, while themes such as patient autonomy and decision-making capacity were present, they were not overtly emphasized, nor was the role of the patient’s wife clearly delineated. Nevertheless, the case was not ethically neutral—it foregrounded issues such as autonomy and the care of patients without acute medical needs, which naturally prompted reflection and discussion. In contrast, topics like side effects, hierarchical dynamics, or threats and violence were less likely to emerge. This openness can be seen as a strength, as it allowed participants to focus on what they perceived as ethically significant. Such interpretive space supports the development of ethical competence by fostering critical reflection and engagement with complex care situations. The case’s portrayal of a multifaceted care scenario may also have contributed to the diversity of suggested action alternatives. It is important to note that the findings reflect the perspectives of experienced ACs. Given that education, training, and professional roles vary across countries, the transferability of results may be limited. However, the findings are likely relevant to similar contexts—particularly those where autonomy and self-determination are central, and where ACs have comparable responsibilities to those in Sweden. While the ethical insights may resonate beyond the prehospital setting, our aim is not to generalize them directly. Rather, we emphasize the importance of enabling healthcare professionals to develop their own ethical insights. We advocate for the use of ECR as a valuable method for identifying and discussing ethical challenges in professional practice and for strengthening ethical competence across diverse nursing contexts—especially through a focus on understanding and responding to the patient’s narrative.

### Limitations

Even though the results provide interesting insights into professional competencies, our methodology does not allow us to say whether the suggested alternatives during the ECRs reflect how participants really act in daily practice. Hence, to the extent our study showcases competencies, it does so in the sense that a range of competencies, tacit and explicit, are necessary to be able to present and discuss alternatives in the way participants did. Whether they also *use* these competencies is, again, a whole different matter. In terms of the authors’ preunderstanding, all the authors except the last author worked in the AS, whereas the last author has extensive experience in conducting ECR work and using the 6SM. In this project, the authors tried to manage the risk of bias in the research process through continuous critical reflection within the author group.

It should be noted that the exact number of invited participants is uncertain, as the email distribution lists used for recruitment included individuals who were on long-term sick leave, parental leave, or other types of extended leave.

## Conclusion

This study highlights the value of ECR as a structured method for supporting in navigating ethical challenges in caring. Ethical competence in the AS context is shaped by context-sensitive decision-making, relational understanding, and professional discretion—an expression of care that is not only technically sound but also humanly meaningful. The results also reflect the challenges of finding the appropriate level of care based on the patient’s needs and wishes in relation to available care options. By providing a “building blocks” approach and specific action alternatives, the combined picture from all ECRs provides a compelling picture of holistic care and uncovers tacit caring competence among ACs. Participating in ECR holds promise for strengthening ethical practice by making implicit knowledge and values explicit. Further research is warranted to explore the ethical relevance of the “building blocks” approach and the specification of action alternatives should be investigated further, along with the capacity for ECR to articulate tacit competences and attitudes toward caring. Moreover, future research is needed to explicitly relate the generated action alternatives (Step V) to the evaluative dimension of ECR (Step VI), to better understand how value-based reasoning may influence decisions and ethical outcomes in AS.

## Data Availability

Data supporting these findings are not publicly available due to the participants’ privacy, the General Data Protection Regulation (GDPR) law, and the conditions of ethical approval from the Swedish Ethical Review Authority. The video is copyrighted and has not been used for other purposes than the present research project. The spoken language in the video is Swedish. The video can be made available by special request.
